# First cardiovascular event in patients with type 2 diabetes mellitus of a cardiovascular risk management program of a poor Colombian population: a cohort study

**DOI:** 10.1186/s12872-018-0993-z

**Published:** 2019-01-08

**Authors:** Pablo Miranda-Machado, Fernando Salcedo-Mejía, Justo Paz Wilches, Juan Fernandez-Mercado, Fernando De la Hoz-Restrepo, Nelson Alvis-Guzmán

**Affiliations:** 1 ALZAK Foundation, 130002, Crespo 70 #6-99, Cartagena, Colombia; 2 Mutual SER E.P.S., Cartagena, Colombia; 3 Universidad Nacional de Colombia, Bogotá, Colombia; 4 Universidad de Cartagena, Cartagena, Colombia

**Keywords:** Cardiovascular outcomes, Diabetes, Poor population

## Abstract

**Background:**

According to several studies in population of high-income countries (HIC), patients with Type 2 diabetes mellitus (DM) have a considerably higher risk of cardiovascular morbidity and mortality. However, it is not clear if the magnitude of this association can be widespread in other populations. The objective of this study was to determine the independent association between Type 2 DM and first cardiovascular event in Colombian Caribbean poor population with no records of previous cardiovascular events reported.

**Methods:**

We retrospectively reviewed the individual records from the hospitalizations database of 64,668 patients of cardiovascular risk management program from July 2014 to December 2015. We used a propensity score matching cohort analysis for this study. The *Kaplan–Meier* curves were constructed for the cardiovascular events related endpoints and matched *Cox-regression* analysis to estimate associations of a history of Type 2 DM with cardiovascular outcomes during 1.5 years of follow-up. A formal sensitivity analysis using *The Breslow-Day and Tarone Homogeneity tests* was conducted.

**Results:**

Out of 56,351 patients with no previous cardiovascular events records, 19,368 (34.4%) patients were found to suffer Type 2 DM. Using propensity scores for Type 2 DM, we gathered a cohort of 18,449 pairs of patients with and without Type 2 DM who were balanced on 22 baseline characteristics. A first cardiovascular event occurred in 650 (3.5%) and 403 (2.1%) matched patients with and without Type 2 DM, respectively, during 1.5 years of follow-up. Type 2 DM was associated with first cardiovascular event (HR 1.69; 95% CI 1.43–2.00; *p* = 0.000), AMI (HR 1.79; 95% CI 1.45–2.20; p = 0.000) and stroke (HR 1.54; 95% CI 1.18–2.02; *p* = 0.001). Hazard ratios (95% CIs) for the association of Type 2 DM with all-cause mortality, cardiovascular mortality and all-cause hospitalization were 1.36 (1.21–1.53; *p* < 0.001), 1.52 (1.12–2.08; p 0.004), and 1.20 (1.21–1.53; p < 0.001), respectively.

**Conclusion:**

Type 2 DM resulted to be a significant independent risk factor for first cardiovascular event in Colombian Caribbean poor population with no previous records of cardiovascular events.

## Background

Diabetes mellitus (DM) is responsible for high global mortality rates and high disability burden [1] and is a major risk factor for cardiovascular disease. The presence of both DM and cardiovascular disease (CVD) increases the risk of death [2–5]. The global prevalence of DM has almost doubled in the adult population since 1980 (4.7 to 8.5%). In 2012, 1.5 million people died by DM. High blood glucose levels caused an additional 2.2 million deaths due to increased cardiovascular risk and other diseases. The percentage of deaths attributable to high blood glucose or diabetes that occurs prior to age 70 is higher in low- and middle-income countries (LMICs) than in high-income countries (HICs) [6].

In the world, 80% of cases of noncommunicable diseases (NCDs) occur in LMICs [7]. Most countries in Latin America, at least during the last 50 years, have experienced an epidemiological transition within a “prolonged polarized model”, presenting complex transformation of their conditions of health, with the existence of a double burden of disease or epidemiological superposition [8, 9]. In Colombia, DM is among the first 10 causes of general mortality and within the first 20 causes of years of healthy life lost (YHLL) in a population ≥ 45 years [10]. According to several studies in population of high-income countries (HIC), patients with Type 2 DM have a considerably higher risk of cardiovascular morbidity and mortality. However, it is not clear if the magnitude of this association can be widespread in other populations. This has been attributed to older age and a higher prevalence of cardiovascular risk factors among people with diabetes [11]. In observational studies of cases and controls that included participants from Latin America, including Colombia, an association between DM and AMI has been reported. In the INTERHEART study, a significant association between DM and AMI (Odds Ratio (OR) 2.59, 95% Confidence Interval (CI) 2.09–3.22) and a population risk attributable to DM of 17.2% was reported [12]. In the INTERSTROKE study, a significant association between DM and stroke (Relative Risk (RR) 1.36, 95% CI 1.10–1.68) and a population risk attributable to DM of 5% was reported [13]. In randomized clinical trials such as the NAVIGATOR study, Latin American participants with glucose intolerance had an increased risk of cardiovascular death (Hazard Risk (HR) 2.68, 95% CI 1.82–3.96) and composite cardiovascular outcome (HR 1.48, 95% CI 1.15–1.92) [14]. Traditional multivariate risk adjustment models may be limited by residual biases and questioned the reliability and when the data are recorded in the context of a clinical trial, they may not reflect the real characteristics of the population by the specified selection criteria. In addition, there may be variation in the management plans of cardiovascular risk programs even within a given region. Matching propensity scores allow balancing the covariates of baselines measured in the cohorts to obtain more robust results [15].

The objective of this study was to examine the association between Type 2 DM with first cardiovascular event in a cohort of type 2 DM Colombian Caribbean poor population with no previous records of cardiovascular events compared with a paired matched cohort of patients with no presence of DM.

## Methods

### Study population and cohort

In 2015 Colombia had 48.2 million people. 10.3 million of them live in the Caribbean Region. For this study, patients from a Colombian public health insurance company were selected. By 2015, it had 1.2 million insured people from poor households of the Colombian Caribbean region (11.6% of the population in the Caribbean region). 49% are male, sex ratio 1:1 and 54% are under 30 years old, which indicates a representative and very similar population structure to the Colombian Caribbean region [16].

The insurance company have a cardiovascular risk management program, which performs screening of cardiovascular risk factors and the detection of cardiovascular events in the affiliated population. Screened patients who reported presence of risk factors were enrolled in the cardiovascular risk management program, which carry out a plan of follow-up activities according to the stratification of cardiovascular risk from the Framingham Risk Score.

Sociodemographic and clinical characteristics of enrolled patients from the program are registered in a private platform of the health insurance company. For the current investigation, we had access to the individual records of service provision registered between July 2014 and December 2015. In 2015, there were 64,668 (66.5% women) patients enrolled in the program. More than 90% of them were over 45 years old.

### History of type 2 diabetes mellitus

Patients with type 2 DM were identified through at least once of the following characteristics: having ICD − 10 diagnosis code, having a personal reported history of DM, having glycosylated hemoglobin levels> 6.5% at least once in records and those under anti-diabetic treatment. From the identified patients, we excluded the ones with a personal history of previous cardiovascular events, and diabetics younger than 30 years under treatment with insulin and without oral anti-diabetics.

### Study outcomes

For the current analysis, the main outcome were first CVD events related endpoints: such as acute myocardial infarction, angina pectoris, acute stroke, mortality due to CVD, and hospitalization due to CVD during 1.5 years of follow-up (12,7 average months of follow-up and range, 1–18 months). CVD events were confirmed by ICD-10 diagnosis CVD events related endpoints from the cardiovascular risk management program hospitalizations database. Follow-up was censored with presence of cardiovascular events, death, or the end of the study, whichever occurred first.

For the current investigation, we had access to hospitalizations records between July 2014 and December 2015, which were previously verified by the internal auditors of the health insurance company. The exact date of death of the patients was not available. So the date of hospitalization due to cardiovascular events or any other date was considered and imputed as the date of death in patients registered as “deceased” in the database of the program whose date of hospitalization was after the date of the last monitoring control observed. In the patients registered as “deceased” who did not have a record of hospitalizations, the median date between the date of the last control observed and the expected date of the next control was considered as the date of death.

### Covariates and propensity score matching

We used the propensity score method and performed 1:1 nearest-neighbor matching without replacement due to imbalances in baseline characteristics between patients with and without Type 2 DM. We used a non-parsimonious multivariable logistic regression model to estimate propensity scores for Type 2 DM. In the model, Type 2 DM was the dependent variable and clinically relevant baseline characteristics were used as covariates. Then a *P* value of less 0.20 was defined for selecting variables for entry into the final model. Selected variables were as follows: age, sex, smoking, physical activity, hypertension, obesity, hyperlipidemia, chronic kidney disease (CKD), hypertension control, Angiotensin-Converting Enzyme Inhibitors (ACEI)-Angiotensin-Receptor Blocker (ARB), calcium-antagonists, statins, antiplatelet and number of anti-hypertensive drugs. By using these covariates, a propensity score was calculated for each patient. Finally, each patient who underwent Type 2 DM was matched to one patient who underwent no Type 2 DM with the closest propensity score. The maximum difference of propensity score for a match was less than 0.03. To ensure that the post-match comparisons between patients with and without diabetes were not affected by the small sample size of the matched cohort, we assembled a pre-match cohort of the same sample size as that of the matched cohort. This was done by first identifying the 18,449 patients with diabetes in the matched cohort. Then, we identified a random sample of 18,449 patients without diabetes from the entire pre-match sample of 36,898 patients without diabetes. Finally, we linked these two data sets, thus assembling a cohort of 18,449 pairs of patients with and without diabetes.

### Statistical analysis

Baseline characteristics were presented through absolute and relative frequencies and means with standard deviations. We used *Pearson Chi-square* and *Wilcoxon Rank-Sum* tests for the pre-match comparison and *paired sample t-test* for the post-match comparisons of baseline covariates between patients with and without DM. The *Kaplan–Meier* curves were constructed for the cardiovascular events related endpoints and matched *Cox-regression* analysis to estimate associations of a history of Type 2 DM with cardiovascular outcomes during 1.5 years of follow-up. Log-minus-log scale risk plots were used to check proportional hazards assumptions. A formal sensitivity analysis using *The Breslow-Day and Tarone Homogeneity tests* was conducted. All statistical analyses were conducted using the Stata version 14.2 software (StataCorp, College Station, TX) and R version 3.4.3 (R Core Team, R Foundation for Statistical Computing). *P* value < 0.05 was considered statistically significant for all tests.

## Results

### Baseline characteristics before propensity matching

All 56,351 patients included in the study are from a low socioeconomic level. We could not access to information on educational level, civil status and other sociodemographic characteristics due to restrictions of the insurance company. After identification, 19,368 (34.4%) patients were identified through the Type 2 DM records as follow; 14,131 (73%) by the ICD-10 diagnosis of DM, 323 (1.7%) by a personal history of DM, 2334 (12.1%) by glycosylated hemoglobin levels> 6.5% at least once and 2579 (13.3%) by the reported treatment with anti-diabetic medicines. Patients were significantly younger in the personal history of Type 2 DM (−) group compared with the personal history of Type 2 DM (+) group (63.6 ± 13.1 years old versus 65.6 ± 13.6 years old); the number of anti-hypertensive drugs in the personal history of Type 2 DM (+) group is lower compared with the personal history of Type 2 DM (−) group (0.69 ± 0.45 versus 0.72 ± 0.44). 36.7% achieved good metabolic control (glycosylated hemoglobin < 7%).

The incidence rate of first cardiovascular event in > 65 years old was 22.9/1000 person – year (95% CI 21.0–25.0) (Incidence Rate Ratio (IRR) 3.15 when ≥ 65 years old group was compared with < 65 years old group, 95% IC 2.65–3.75). The incidence rate of first acute myocardial infarction or angina pectoris event (AMI) in > 65 years old was 13.9/1000 person – year (95% CI 12.4–15.5) (IRR) 2.63 when ≥ 65 years old group was compared with < 65 years old group, 95% IC 2.14–3.26). The incidence rate stroke in > 65 years old was 9.31/1000 person – year (95% CI 8.13–10.67) (IRR 4.68 when ≥ 65 years old group was compared with < 65 years old group, 95% IC 3.41–6.53).

There were several parameters of baseline characteristics statistically higher in the family history of DM (+) group, including the percentage of female gender (68.07% vs. 65.33%), hypertension control (90.74% vs. 89.28%), use of antiplatelet (41.59% vs. 37.12%) and use of statins (52.05% vs. 44.54%). The incidence rate of first cardiovascular event in man was 20.9/1000 person – year (95% CI 18.6–23.5) (IRR 1.49 when men’s group was compared with women’s group, 95% IC 1.29–1.72). The incidence rate of AMI in man was 14.2/1000 person – year (95% CI 13.3–16.3) (IRR 1.66 when men’s group was compared with women’s group, 95% IC 1.38–1.99). The incidence rate of stroke in man was 6.95/1000 person – year (95% CI 5.67–8.51) (IRR 1.23 when men’s group was compared with women’s group, 95% IC 0.96–1.67).

Among 19,368 subjects with a personal history of Type 2 DM (+) group, 667 (3.4%) subjects had a first cardiovascular event before propensity score matching. Among 18,449 subjects with a personal history of Type 2 DM (+) group, 650 (3.5%) subjects had a first cardiovascular event after propensity score matching. The incidence rate of first cardiovascular event was 22.2/1000 person – year (95% CI 19.9–24.7) (IRR 1.86 when Type 2 DM (+) group was compared with Type 2 DM (−) group, 95% IC 1.65–2.15). The incidence rate of AMI was 14.4/1000 person – year (95% CI 12.6–16.4) (IRR 2.00 when Type 2 DM (+) group was compared with Type 2 DM (−) group, 95% IC 1.67–2.39). The incidence rate of stroke was 8.05/1000 person – year (95% CI 6.73–9.63) (IRR 1.66 when Type 2 DM (+) group was compared with Type 2 DM (−) group, 95% IC 1.31–2.11).

Among 19,368 subjects with a personal history of Type 2 DM (+) group, 3418 (17.4%) subjects were under treatment of human or analogous insulin. Of these, 213 (6.2%) subjects had a first cardiovascular event before propensity score matching. The incidence rate of first cardiovascular event was 41.01/1000 person – year (95% CI 34.08–49.36) (IRR 2.05 when Type 2 DM (+) group under human or analogous insulin treatment was compared with Type 2 DM (+) group without treatment with human or analogous insulin, 95% IC 1.73–2.42). The incidence rate AMI was 27.4/1000 person – year (95% CI 21.90–34.44) (IRR 2.03 when Type 2 DM (+) group under human or analogous insulin treatment was compared with Type 2 DM (+) group without treatment with human or analogous insulin, 95% IC 1.65–2.50). The incidence rate of stroke was 14.64/1000 person – year (95% CI 10.74–19.97) (IRR 2.13 when Type 2 DM (+) group under human or analogous insulin treatment was compared with Type 2 DM (+) group without treatment with human or analogous insulin, 95% IC 1.60–2.80).

**Fig. 1:**
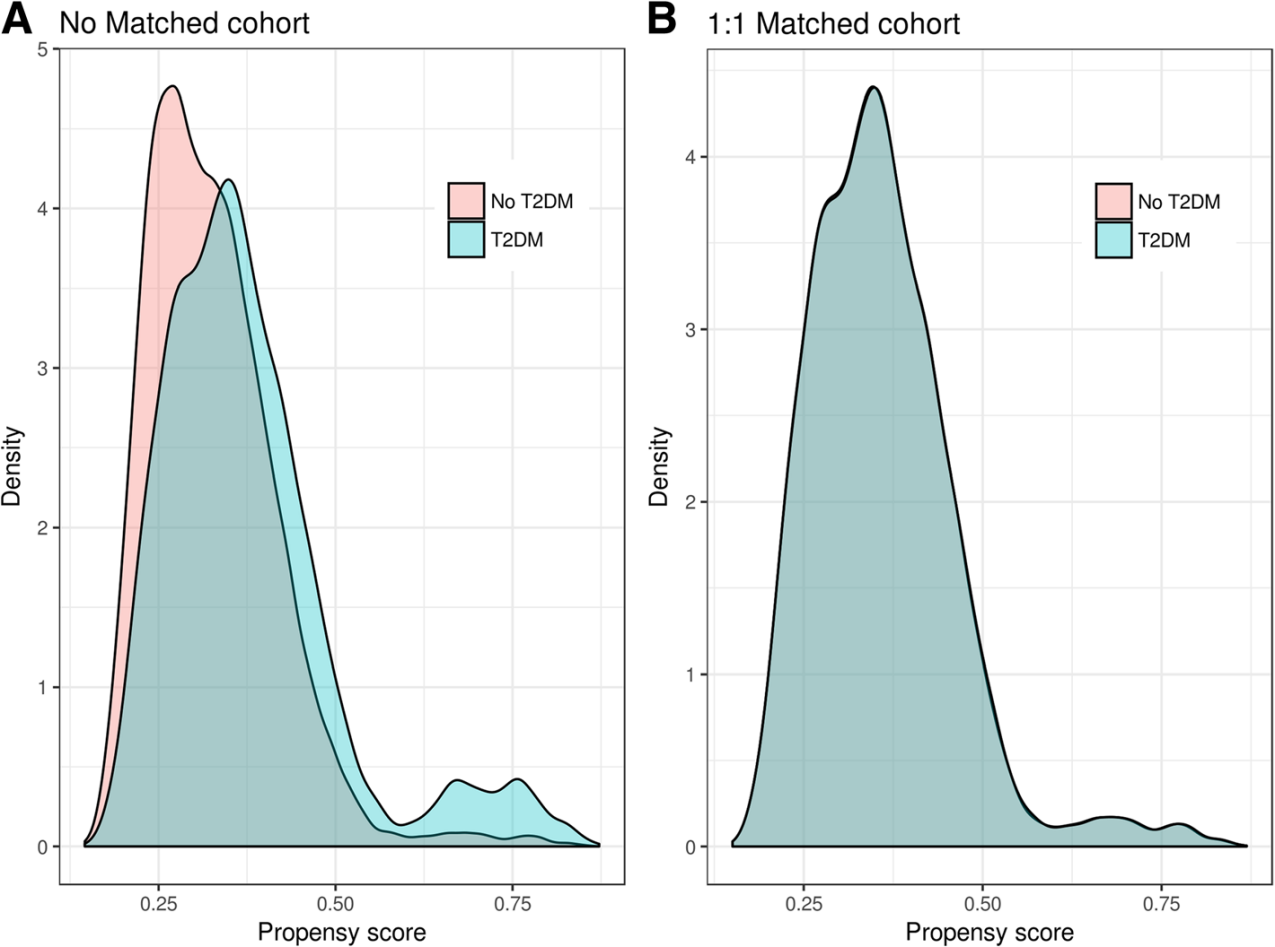
Propensity score distribution before (**a**-No Matchet cohort) and after (**b**-Matchet cohort) propensity score matching

To explore this imbalance, we illustrate a histogram of propensity score distribution for groups with and without personal history Type 2 DM before Fig. 1 (a) and after Fig. 1 (b) propensity matching.

### Baseline characteristics after Propensity matching

According to the propensity score matching 1:1 shown in Table 1, 18,446 patients in the personal history of Type 2 DM (+) group were matched with 18,446 in the personal history of Type 2 DM (−) group. All absolute standardized differences after the match for all covariates were < 5% and showed sufficient overlap in the estimated propensity scores. The characteristics of propensity score-matched patients with (*n* = 18,449) and without (*n* = 18,449) Type 2 DM are shown in Table 1.

**Table 1:** Baseline Characteristics by history of Type 2 DM before and after propensity Matching cohort

	Before propensity matching (*N* = 56,351)			After propensity matching (*N* = 36,898)		
n(%) / mean(±sd)	No T2DM	T2DM	P	No T2DM	T2DM	P
Women	24,495	13,299	< 0.001	12,741	12,644	0.276
	66.23	68.66		69.06	68.53	
Age	65.50 (+ − 13.66)	63.24 (+ − 13.08)	< 0.001	63.7 (+ − 13.5)	63.8 (+ − 13.0)	0.7186
Hypertension	36,444	17,998	< 0.001	17,915	17,935	0.531
	98.54	92.93		97.11	97.21	
Dyslipidemia	31,323	16,685	< 0.001	15,956	15,983	0.680
	84.7	86.15		86.49	86.63	
Obesity	14,323	9700	< 0.001	9278	9315	0.700
	38.73	50.08		50.29	50.49	
Controlled Hypertension	33,096	17,582	< 0.001	16,652	16,702	0.377
	89.49	90.78		90.26	90.53	
CKD	5440	3184	< 0.001	3040	3034	0.933
	14.71	16.44		16.48	16.45	
Number of antihypertensive drugs	0.72 (+ − 0.44)	0.67 (+ − 0.46)	< 0.001	1.81(+ − 1.66)	1.82(+ − 1.8)	0.227
ACEI-ARB	24,218	12,126	< 0.001	12,075	12,072	0.974
	65.48	62.61		65.45	65.43	
Antiplatelet	12,957	7492	< 0.001	7242	7347	0.264
	35.04	38.68		39.25	39.82	
Calcium Antagonists	9943	5878	< 0.001	5776	5817	0.646
	26.89	30.35		31.31	31.53	
Statins	15,624	9672	< 0.001	9323	9382	0.539
	42.25	49.94		50.53	50.85	
Insulin	0	3418	< 0.001	0	3222	< 0.001
	0	17.65		0	17.46	
Human Insulin	0	1604	< 0.001	0	1497	< 0.001
	0	8.28		0	8.11	
Analogous Insulin	0	2503	< 0.001	0	2375	< 0.001
	0	12.92		0	12.87	
Sulfonylureas	0	5852	< 0.001	0	5548	< 0.001
	0	30.21		0	30.07	
Metformin	0	8430	< 0.001	0	7950	< 0.0010
	0	43.53		0	43.09	
DPP4-I	0	677	< 0.001	0	648	< 0.001
	0	3.5		0	3.51	
GLP1 receptor agonists	0	88	< 0.001	0	85	< 0.001
	0	0.45		0	0.46	

### Type 2 DM and first CVD Events

**Fig. 2:**
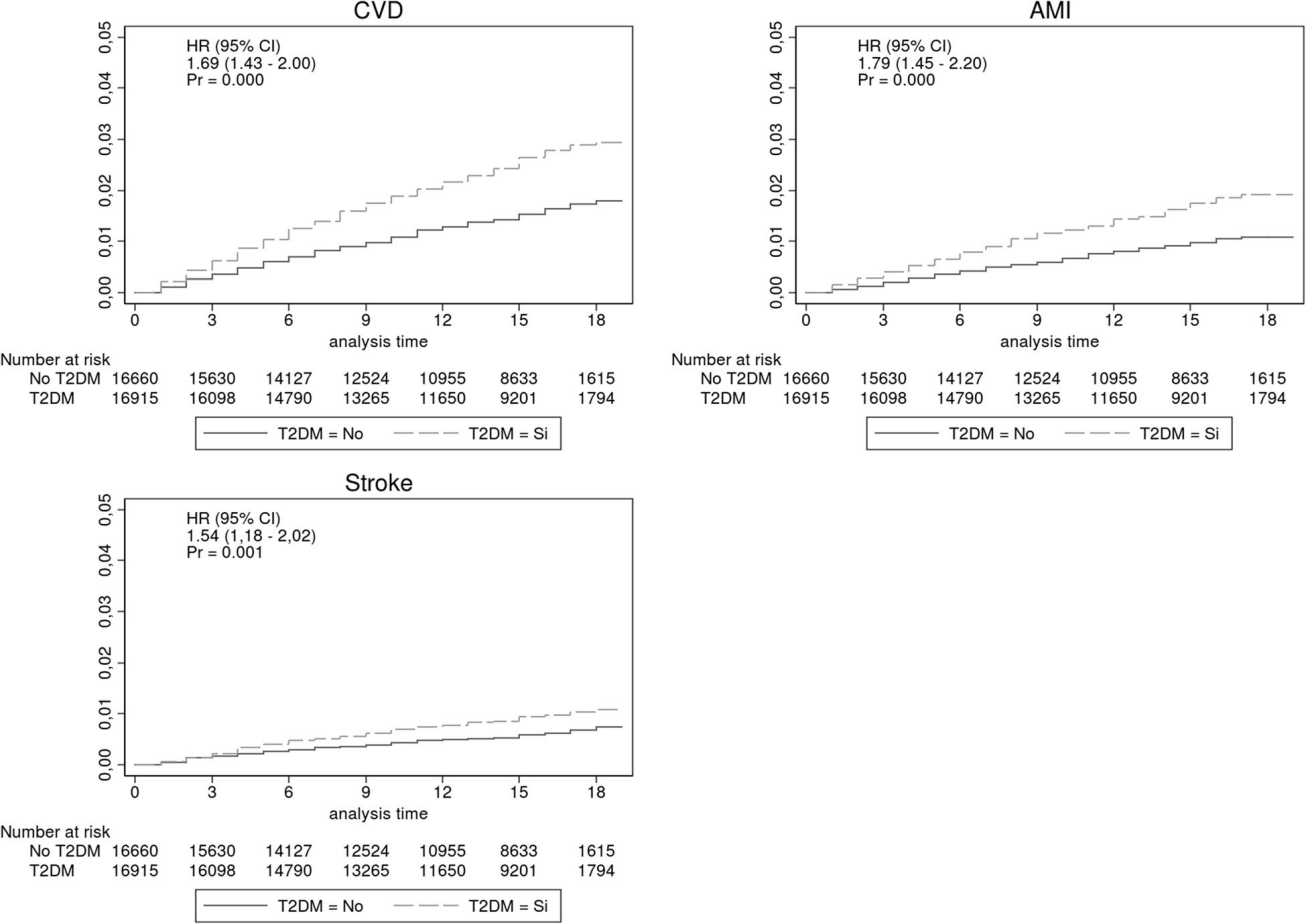
Kaplan-Meier plots for first cardiovascular event in T2DM matching cohort

In the group with and without a personal history of DM type 2, the first CVD event occurred in 650 (3.5%) and 403 (2.1%) patients, respectively (incidence rate 22.1/1000 person – year (95% IC 19.8–24.6) and HR 1.69; 95% CI 1.43–2.00; Fig. 2). In the subgroups analysis, the homogeneous association that a personal history of DM type 2 had with the first CVD event in a broad spectrum of patients was estimated (Table 2).

**Table 2:** Association of a history of Type 2 DM and first event cardiovascular

	T2DM (event/N)					Effect	Breslow-Day homogeneity test
	No	Yes	OR	IC95			
Sex							
Women	207/12741	370/12644	1.825	1.532	2.176	0.452	0.056
Men	196/5708	280/5805	1.425	1.178	1.725	0.298	
Age							
< 65 years	136/9807	196/9719	1.464	1.168	1.838	0.317	0.238
> =65 years	267/8642	454/8730	1.721	1.471	2.014	0.419	
CKD							
No	308/15409	503/15415	1.654	1.429	1.914	0.395	0.760
Yes	95/3040	147/3034	1.578	1.205	2.076	0.366	
Controlled Hypertension							
No	47/1797	76/1747	1.693	1.154	2.505	0.409	0.847
Yes	356/16652	574/16702	1.629	1.422	1.867	0.386	
Dyslipidemia							
No	18/2493	33/2466	1.865	1.016	3.526	0.464	0.647
Yes	385/15956	617/15983	1.624	1.424	1.852	0.384	
Obesity							
No	242/9171	385/9134	1.624	1.374	1.918	0.384	0.873
Yes	161/9278	265/9315	1.658	1.354	2.034	0.397	
Hypertension							
No	1/534	0/514					0.2095^a^
Yes	402/17915	650/17935	1.638	1.441	1.862	0.390	
ACEI-ARB							
No	37/6374	47/6377	1.272	0.808	2.015	0.214	0.225
Yes	366/12075	603/12072	1.682	1.470	1.924	0.405	
Calcium Antagonist							
No	169/12673	269/12632	1.610	1.320	1.964	0.379	0.815
Yes	234/5776	381/5817	1.660	1.400	1.969	0.398	
Statins							
No	61/9126	92/9067	1.523	1.089	2.143	0.344	0.632
Yes	342/9323	558/9382	1.661	1.444	1.910	0.398	
Antiplatelet							
No	95/11207	144/11102	1.537	1.176	2.016	0.349	0.599
Yes	308/7242	506/7347	1.665	1.436	1.931	0.399	
Number antihypertensive drugs							
< 2	49/9062	68/9421	1.337	0.912	1.973	0.252	0.172
> =2	354/9387	582/9028	1.758	1.532	2.018	0.431	

^a^ Tarone’s homogeneity test

In the group with and without a personal history of DM type 2, the first acute myocardial infarction or angina pectoris event (AMI) occurred in 424 (2.3%) and 254 (1.3%) patients, respectively (incidence rate 14.4 person -year (95% CI 12.6–16.5); HR 1.79; 95% CI 1.45–2.20; *p* = 0.000; Fig. 2 and Table 3). In the group with and without a personal history of DM type 2, the first acute stroke event occurred in 234 (1.2%) and 153 (0.8%) patients, respectively (incidence rate 8,1 / 1000 person – year (95% CI 6,7 - 9,6); HR 1.54; 95% CI 1.18–2.02; *p* = 0.001; Fig. 2 and Table 4).

**Table 3:** Association of a history of Type 2 DM and first event AMI

	T2DM (event/N)					Effect	Breslow-Day homogeneity test
	No	Yes	OR	IC95			
Sex							
Women	133/12741	228/12644	1.741	1.397	2.175	0.426	0.636
Men	133/5708	228/5805	1.613	1.276	2.047	0.380	
Age							
< 65 years	100/9807	144/9719	1.460	1.121	1.906	0.315	0.176
> =65 years	154/8642	280/8730	1.826	1.491	2.243	0.452	
CKD							
No	198/15409	330/15415	1.681	1.402	2.015	0.405	0.944
Yes	56/3040	94/3034	1.704	1.206	2.424	0.413	
Control HTA							
No	31/1797	48/1747	1.609	0.998	2.629	0.585	0.385
Yes	223/16652	376/16702	1.697	1.431	2.012	0.398	
Dyslipidemia							
No	8/2493	19/2466	2.412	1.006	6.379	0.464	0.647
Yes	246/15956	405/15983	1.660	1.410	1.955	0.384	
Obesity							
No	146/9171	246/9134	1.711	1.385	2.119	0.416	0.835
Yes	108/9278	178/9315	1.654	1.293	2.124	0.395	
Hypertension							
No	1/534	0/514					0.2026^a^
Yes	253/17915	424/17935	1.690	1.440	1.984	0.408	
ACEI-ARB							
No	22/6374	25/6377	1.136	0.614	2.116	0.120	0.159
Yes	232/12075	399/12072	1.745	1.476	2.062	0.427	
Calcium Antagonist							
No	120/12673	191/12632	1.606	1.270	2.038	0.377	0.577
Yes	134/5776	233/5817	1.757	1.410	2.196	0.431	
Statins							
No	25/9126	55/9067	2.222	1.359	3.723	0.550	0.223
Yes	229/9323	369/9382	1.626	1.371	1.929	0.385	
Antiplatelet							
No	44/11207	83/11102	1.911	1.309	2.823	0.477	0.442
Yes	210/7242	341/7347	1.630	1.363	1.949	0.386	
Number antihypertensive drugs							
< 2	24/9062	34/9421	1.364	0.785	2.406	0.267	0.324
> =2	230/9387	390/9028	1.798	1.518	2.129	0.444	

^a^ Tarone’s homogeneity test

**Table 4:** Association of a history of Type 2 DM and first event stroke

	T2DM (event/N)					Effect	Breslow-Day homogeneity test
	No	Si	OR	IC95			
Sex							
Women	78/12741	145/12644	1.883	1.419	2.515	0.469	0.024
Men	75/5708	89/5805	1.169	0.848	1.615	0.145	
Age							
< 65 years	37/9807	53/9719	1.448	0.933	2.269	0.309	0.769
> =65 years	116/8642	181/8730	1.556	1.223	1.986	0.357	
CKD							
No	112/15409	179/15415	1.605	1.259	2.053	0.377	0.474
Yes	41/3040	55/3034	1.350	0.882	2.082	0.260	
Control HTA							
No	17/1797	29/1747	1.767	0.935	3.440	0.434	0.629
Yes	136/16652	205/16702	1.509	1.208	1.890	0.337	
Dyslipidemia							
No	10/2493	15/2466	1.520	0.637	3.790	0.342	0.980
Yes	143/15956	219/15983	1.536	1.237	1.912	0.349	
Obesity							
No	99/9171	145/9134	1.478	1.135	1.931	0.323	0.617
Yes	54/9278	89/9315	1.648	1.160	2.357	0.393	
Hypertension							
No	0/534	0/514					1^a^
Yes	153/17915	234/17935	1.535	1.245	1.896	0.348	
ACEI-ARB							
No	16/6374	22/6377	1.376	0.690	2.804	0.273	0.720
Yes	137/12075	212/12072	1.558	1.249	1.948	0.358	
Calcium Antagonist							
No	52/12673	81/12632	1.566	1.091	2.264	0.362	0.886
Yes	101/5776	153/5817	1.518	1.169	1.976	0.341	
Statins							
No	36/9126	38/9067	1.063	0.655	1.726	0.059	0.080
Yes	117/9323	196/9382	1.679	1.326	2.133	0.404	
Antiplatelet							
No	52/11207	63/11102	1.224	0.834	1.804	0.183	0.159
Yes	101/7242	171/7347	1.685	1.307	2.181	0.406	
Number antihypertensive drugs							
< 2	26/9062	34/9421	1.259	0.733	2.186	0.206	0.340
> =2	127/9387	200/9028	1.652	1.313	2.084	0.395	

^a^ Tarone’s homogeneity test

### Type 2 DM and other outcomes

Significant unadjusted associations were estimated between the personal history of DM type 2 and several outcomes among the pre-match cohort. Among the 36,898 balanced cohorts, type 2 DM was associated with an increased risk of hospitalization from any cause and association with cardiovascular death, stroke death, and death from all causes (Table 5).

**Table 5:** Effect of a history of Type 2 DM and other outcomes

(N / %)	NO T2DM	T2DM	OR	IC95%		P
Before matching						
All-cause Hospitalization	4638 (12.54)	4001 (20.66)	1.816	1.733	1.903	< 0.001
Cardiovascular hospitalization	707 (15.24)	667 (16.67)	1.112	0.989	1.251	0.071
AMI hospitalization	432 (9.31)	434 (10.85)	1.185	1.027	1.367	0.018
Stroke hospitalization	280 (6.04)	241 (6.02)	0.998	0.832	1.196	0.979
All – cause death	1077 (2.91)	684 (3.53)	1.220	1.106	1.347	< 0.001
Cardiovascular death	168 (15.60)	157 (22.95)	1.612	1.255	2.069	< 0.001
AMI hospitalization	94 (8.73)	98 (14.33)	1.749	1.279	2.390	< 0.001
Stroke hospitalization	76 (7.06)	62 (9.06)	1.313	0.909	1.890	0.127
After matching						
All-cause hospitalization	2443 (13.24)	3865 (20.95)	1.736	1.642	1.836	< 0.001
Cardiovascular hospitalization	403 (16.5)	650 (16.82)	1.023	0.891	1.175	0.739
AMI hospitalization	254 (10.4)	424 (10.97)	1.062	0.898	1.256	0.474
Stroke hospitalization	153 (6.26)	234 (6.05)	0.965	0.778	1.199	0.737
All-cause death	500 (2.71)	676 (3.66)	1.365	1.212	1.538	< 0.001
Cardiovascular death	82 (16.4)	156 (23.08)	1.529	1.126	2.085	0.005
AMI hospitalization	46 (9.20)	98 (14.5)	1.673	1.140	2.483	0.006
Stroke hospitalization	38 (7.6)	61 (9.02)	1.206	0.776	1.893	0.385

## Discussion

The data from the *Framingham Heart Study* made it possible to identify DM as an important cardiovascular risk factor, mainly in women [17]. To the best of our knowledge, this is the first report of an association between Type 2 DM and first cardiovascular events in a propensity-matched cohort of poor population without a history of previous cardiovascular events in Latin America and Colombia. According to several studies, all patients with DM can be treated as if they had prior cardiovascular disease since the risk of fatal AMI in patients with DM without previous AMI is similar to that of patients without DM who have survived an AMI [11, 17]. In the study of Becker et al., 10-year follow-up, women with DM, but without prior cardiovascular disease have a risk of cardiovascular events that is similar to that of women without diabetes but with prior cardiovascular disease, whereas in men the presence of prior cardiovascular disease conferred a higher risk [11]. In our study, in the pre-matched cohort, the risk of the first cardiovascular event was significantly higher in women with Type 2 DM. In the post-matched cohort, these differences by sex disappeared for almost all outcomes, except for the stroke incidence that was significantly higher in women with Type 2 DM. In the *Framingham Heart Study* for stroke, women with DM had a higher incidence than men did with DM [17].

The findings of the current analysis demonstrate that in patients without a personal history of previous cardiovascular events, a history of Type 2 DM was associated with an increased risk of a first cardiovascular event, which was primarily driven by an increase in AMI. Type 2 DM was also associated with risk of all-cause hospitalization, cardiovascular death, stroke death and all-cause death, but had no independent association with cardiovascular hospitalization. In the *Framingham Heart Study,* an increased mortality due to CVD in patients with DM compared with patients without DM was reported [17]. In the INTERHEART study, a significant association between DM and AMI (OR 2.59, 95% CI 2.09–3.22) and a population risk attributable to DM of 17.2% was reported [12]. In the INTERSTROKE study, a significant association between DM and stroke (RR 1.36, 95% CI 1.10–1.68) and a population risk attributable to DM of 5% was reported [13]. These results should be interpreted considering the limitations and possible biases of a case-control study, in which the presence of exposure variables is determined after the onset of the disease [18]. In randomized clinical trials such as the NAVIGATOR study, Latin American participants with glucose intolerance had an increased risk of cardiovascular death (HR 2.68, 95% CI 1.82–3.96) and composite cardiovascular outcome (HR 1.48, 95% CI 1.15–1.92). When the data are recorded in the context of a clinical trial, they may not reflect the real characteristics of the population by the specified selection criteria [14]. In the PURE study identified that although risk-factor burden may be lower in LMIC, the risk of cardiovascular events was much higher [19]. In the current study, the risk of first cardiovascular event was significantly higher in the Type 2 DM group (HR 1.69; 95% CI 1.43–2.00).

In Colombia, DM is among the first 10 causes of general mortality and within the first 20 causes of years of healthy life lost (YHLL) in a population ≥ 45 years [10]. In Colombia, according to data from individual health care records (RIPS) and systematic reviews between 2010 and 2014, approximately 1,500,000 patients with type 2 DM were estimated [10]. According to the National Department of Statistics (DANE) of Colombia, between January 2016 and August 2018, 60,944 deaths from ischemic heart disease (17.1%), 24,548 deaths from cerebrovascular diseases (6.9%) and 11,743 deaths from DM (3.3%), were estimated. According to the cumulative incidence of first cardiovascular events (3.5%) and cardiovascular mortality (22.9%) in Type 2 DM group (+) estimated in the current study, approximately 52,500 first-time cardiovascular events and around 12,000 cardiovascular deaths per year would be expected to occur. These estimates are similar to the annual deaths due to DM estimated by the International Diabetes Federation (IDF) (11,400 deaths due to DM) [6] and the non-fetal death statistics of the DANE of Colombia (11,743 deaths due to DM).

Care of a patient with DM requires a multifactorial approach from the cardiovascular risk management program. All patients are at risk of developing vascular complications of DM, and these risks represents ultimately result in a doubled risk of mortality in patients with DM. Above and beyond targeted interventions, we now know that strict multifactorial interventions can result in a clinically significant reduction in both mortality and cardiovascular disease. These conclusions provide significant information on the importance of Type 2 DM in increasing the risk of first cardiovascular events in the poor population of the Colombian Caribbean region and the importance of an adequate diagnosis and treatment of Type 2 DM. However, evidence that an adequate metabolic control reduces the rates of cardiovascular events and death is not clear, although a discrete cardiovascular benefit may be observed after a prolonged follow-up period [20–24]. Evidence from HICs indicates that multiple risk factor intervention programmes do not result in reductions in cardiovascular events but may be effective in reducing mortality in high-risk hypertensive and diabetic populations. Due to the differences in the structure of the communities and the target population, caution is needed to generalize the results to LMICs. In the systematic review of Uthman et al., evidence about effectiveness of multiple risk factor interventions (with or without pharmacological treatment) in LMICs was scarce and only one study reported cardiovascular outcomes and multiple risk factor interventions did not reduce the incidence of cardiovascular events and none of the included trials reported all-cause mortality [25]. For cardiovascular risk management programs it is important to evaluate the impact of their interventions over time to achieve optimal primary cardiovascular prevention in patients under the care of the program [26]. There may be variation in the management plans of cardiovascular risk programs even within a given region beyond differences in health budget and system characteristics [27]. The use of statins in the LMIC is low in comparison with the HIC [28]. In the NAVIGATOR study, was reported that patients in Latin America used fewer therapies for prevention of cardiovascular events, such as aspirin (32% versus 47% in North America and 35% in Europe), lipid-lowering therapies (28% versus 55% in North America and 35% in Europe), and ACEIs (4% versus 9% in North America and 8% in Europe) [14]. In the TECOS study, low use of statins and aspirin in Latin America compared to North America was reported [29]. Recently, trials evaluating anti-diabetic drugs (empagliflozin, liraglutide, pioglitazone and semaglutide) have shown improved cardiovascular outcomes in patients with Type 2 DM [30–34]. The findings of the current analysis report a low use of statins, aspirin and new anti-diabetic drugs.

Our study has several strengths and limitations. More than 35 thousand of patients were included and, in spite of the nearly 1000 patients with type 2 DM lost, there were no differences in the incidences and incidence rates between the groups after propensity matching. We tried that the possibility of residual confounding, by the measured covariates and confounding by the unmeasured covariates that explain the associations between Type 2 DM and first cardiovascular event, were unlikely, considering that our patients were matched in 22 baseline characteristics and the conclusions of our analysis of sensitivity suggest that the association between Type 2 DM and a first cardiovascular event was quite insensitive to an unmeasured binary confounder.

Within limitations, we faced restrictions to access to sociodemographic data due to restrictions from the insurance company. In order to identify the diagnosis of cardiovascular outcomes we used the records of previous cardiovascular events and the ICD-10 hospital discharge code. However, we could not have data on confirmatory tests.

On the other hand, the exact date of death of patients and date of enrollment on the cardiovascular risk management program was not available.

We did not have access to information related to the treatment of cardiovascular events such as medications, thrombolysis, percutaneous coronary intervention (PCI), coronary artery bypass surgery (CABG), etc. In conclusion, in the poor population of the Colombian Caribbean without a personal history of cardiovascular events receiving standard therapy, DM produces cardiovascular outcomes. It is not known if a more aggressive screening of DM, DM education at every level, quality care registries and taxes on sugary drinks can reduce the risk of a first cardiovascular event in these patients and should be determined prospectively in future studies in Latin America.

## Abbreviations

AMI: Acute myocardial infarction
CVD: Cardiovascular disease
DANE: Department of Statistics of Colombia
HICs: High-income countries
ICD-10: International statistical classification of diseases and related health problems version 10
LMICs: Low- And Middle-Income Countries
NCDs: Noncommunicable diseases
T2DM: Type 2 diabetes millitus
YHLL: Years of healthy life lost
